# MicroRNAs in pediatric central nervous system embryonal neoplasms: the known unknown

**DOI:** 10.1186/s13045-014-0101-5

**Published:** 2015-02-06

**Authors:** Maria Braoudaki, George I Lambrou

**Affiliations:** First Department of Pediatrics, University of Athens, Choremeio Research Laboratory, Athens, Greece; University Research Institute for the Study and Treatment of Childhood Genetic and Malignant Diseases, University of Athens, Aghia Sophia Children’s Hospital, Athens, Greece

**Keywords:** CNS, Medulloblastoma, AT/RT, Pediatric Embryonal Tumors, miRNA

## Abstract

MicroRNAs (miRNAs) are endogenous short non-coding RNAs that repress post-transcriptional regulation of gene expression, while embryonal central nervous system tumors are the foremost cause of mortality in children suffering from a neoplasm. MiRNAs and their regulatory mechanisms are new to understand, while pediatric CNS tumors are difficult to comprehend. Therefore, identification of the link between them composes a major scientific challenge. The present study, reviewed the current knowledge on the role of miRNA in pediatric CNS embryonal tumors, attempting to collect the existing information in one piece of work that could ideally be used as a guide for future reference and research.

## Introduction

### Introducing microRNAs

MicroRNAs (miRNAs) are endogenous short non-coding RNAs of 19–24 nucleotides in length that repress post-transcriptional regulation of gene expression. Single-stranded miRNAs are predicted to regulate 30% of all genes and to target sequences in the 3’ untranslated region (3’ UTR) of genes. They bind through partial sequence homology to the 3’ UTR of target mature protein coding messenger RNAs (mRNAs) and either inhibit mRNA translation through RNA interference (RNAi) or less frequently induce mRNA degradation. As a result, they impact vital cellular and physiological processes including differentiation, proliferation, growth, stress response, apoptosis and survival [1-3]. It has become increasingly evident that deregulation of microRNAs is implicated in a wide range of serious human diseases such as cardiovascular disease [4-8], neurological disorders [9], immune-mediated disorders [10,11], viral infections [12], diabetes [13,14], obesity [5,15-17], rheumatoid arthritis [13,18,19] and several types of cancer [3,6,14,19-22] including breast cancer [23-26], bladder cancer [27-29], and kidney cancer [30-33]. In general, we do not know much on the functionality and role of miRNAs in pediatric tumors. As a remark, knowledge adds up with time while interpretation of complex information lacks in progress. Yet, this is what makes biological phenomena so challenging and beautiful.

### Oncogenesis in childhood neoplasms

#### Clonal evolution and cancer stem cells

Substantial growing experimental evidence in several malignancies has demonstrated that only a distinct subpopulation of tumor cells, termed cancer stem cells (CSCs), contain the ability to undergo self-renewal and differentiation (properties of normal stem cells). Hence, they have the ability to initiate tumorigenesis and support ongoing tumor growth. Furthermore, it appears that, like their normal stem cell counterparts, CSCs have increased resistance to standard cytotoxic therapies. These findings have coalesced into the cancer stem cell hypothesis of tumorigenesis, which has remarkable implications on our understanding of tumor initiation, disease progression, and treatment response. In general, two major models have been described for tumor propagation: the clonal evolution model and the CSC hypothesis. According to the clonal evolution model, neoplasms arise from a single cell of origin and tumor progression results from acquired genetic variability within the original clone allowing sequential selection of more aggressive sublines. The CSC hypothesis sustains that tumor cells are heterogeneous and only the CSC subset has the ability to proliferate extensively and form new tumors [34-37].

In general, CSCs are highly important to understand the underlying tumor biology or pathogenesis. However, even though there has been suggested that tumor growth is sustained by a subpopulation of cells with stem-like features, little is known on their genomic characterization and their genetic stability.

#### Neural stem cells

The cellular origin of pediatric brain tumors remains unclear. One possibility is that they arise by transformation of proliferating neural stem cells (NSCs), which retain the ability to self-renew and differentiate into neurons and glia. There are several lines of indirect evidence in support of this hypothesis including: a) Pediatric brain tumors often contain multiple cell types suggestive of an origin from a cell with multilineage potential; b) Several pediatric brain tumors appear to arise from the ventricular zone, which is the location of the NSCs; c) Both pediatric brain tumors and NSCs express nestin, an intermediate filament characteristic of several progenitors; d) Pediatric brain tumors frequently express genes that regulate proliferation and self-renewal of normal NSCs whilst mutations in genes that normally regulate neural stem cell proliferation are frequently found in pediatric brain tumors; e) Forced expression of oncogenes in neural stem and progenitors cells in Severe Combined Immunodefficient (SCID) mice produces tumors that are similar to primary human tumors. In models of acute myeloid leukemia for instance, CSCs have been isolated and re-passaged into experimental animals to form novel tumors, providing strong evidence that these cells are the root cause of the tumor [38-43].

#### Controversies

With respect to stem cells, a lot of debate has been done on their role and their functionality in tumorigenesis. Other theories have proposed that cancer could be a “wound that never heals”, which along with the role of the microenvironemt constitute the explosive mixture of the disease. A very interesting issue addressed before concerns the aspect of proliferation, which consists of a tumor trait and regeneration which consists of a stem cell trait [44]. Regeneration is closely linked to proliferation, while the opposite is not always true especially for tumors. Yet, if we examine this aspect from the tumor point of view then its proliferation that coincides with its regeneration. This is a very delicate phenomenon and the perspective of examination might lead to its comprehension. For example, there is a vast difference between theorizing tumors as an abnormal condition and as a normal evolutionary reaction.

### MicroRNAs and Childhood CNS Neoplasms

A search in the Pubmed Database returns a total of ~80 articles with the keywords “*miRNA medulloblastoma*” and nine articles for “*miRNA childhood Central Nervous System Tumors*”. This highlights the novelty of the subject of miRNA role in childhood neoplasms. Childhood neoplasms of the central nervous system (CNS) are rare with an incidence of less than five newly diagnosed cases for every 100,000 births [45]. The discovery of miRNAs has provided a wealth of knowledge regarding clinical approach, diagnosis and treatment. Their potential as specific disease biomarkers and targets of therapeutic intervention holds great promise. Yet, we should keep in mind our former remark that with increasing discovery of elements of the tumor puzzle their comprehension still falls behind.

### Scope

In the present work, we will focus specifically on pediatric central nervous system (CNS) embryonal tumors and the role of miRNAs expression profiles. Herein, the emerging role of miRNAs’ differential expression in the pathogenesis of pediatric central nervous system embryonal tumors and their potential as therapeutic targets is addressed.

## Pediatric CNS embryonal tumors

Pediatric tumors of embryonal origin are a heterogeneous group of malignant neoplasms that comprise by far the largest group of malignant brain tumors in childhood, highly associated with increased mortality and long-term morbidity. On the basis of their morphological, immunohistochemical and molecular features, these tumors are broadly classified by the World Health Organization (WHO). The new WHO classification on brain tumors has established some relevant changes in the group of CNS embryonal neoplasms. The above group of neoplasms is comprised from several brain tumor types including medulloblastoma, CNS primitive neuroectodermal tumors, medulloepithelioma, ependymoblastoma and atypical teratoid rhabdoid tumor/rhabdoid tumor predisposition syndrome [46]. The implicated miRNAs referred in the present work are summarized in Table 1.

**Table 1:** **Summary of miRNAs participating in CNS Embryonal Tumors**

**miRNA**	**miRNA expression pattern**	**Target gene**	**Molecule/gene expression pattern**	**miRNA regulating role with respect to target-molecule/gene**	**miRNA regulating role to MB**	**CNS neoplasm**	**Reference**
**miR-124**	↓ compared to normal brain tissue	CDK6	↑ compared to normal brain tissue	Interacts	Suppresses proliferation, not apoptosis	MB	Pierson J, Hostager B et al. [58]
**miR-124**	↓ compared to normal brain tissue	SLC16A1	↑ compared to normal brain tissue	Suppression	Suppresses proliferation, not apoptosis	MB	Li KKW, Pang JCS et al. [59]
**miR-129**	↓ compared to normal brain tissue	CDK6	↑ compared to normal brain tissue	Suppression	Suppresses proliferation, not apoptosis	MB	Wu J, Qian J et al. [61]
**miR-125b**	↓ compared to normal brain tissue	SMO	Promote cerebellar tumorigenesis	Suppression	Suppresses proliferation,/cell cycle	MB	Ferretti E, De Smaele E et al. [62]
		GLI1	Both ↓ ↑/ Promote cerebellar tumorigenesis	Suppression	Suppresses proliferation/cell cycle	MB	
**miR-324-5p**	↓ compared to normal brain tissue	SMO	Promote cerebellar tumorigenesis	Suppression	Suppresses proliferation,/cell cycle	MB	
		GLI1	Both ↓ ↑/ Promote cerebellar tumorigenesis	Suppression	Suppresses proliferation/cell cycle	MB	
**miR-326**	↓ compared to normal brain tissue	SMO	Promote cerebellar tumorigenesis	Suppression	Suppresses proliferation,/cell cycle	MB	
		GLI1	Both ↓ ↑/ Promote cerebellar tumorigenesis	Suppression	Suppresses proliferation/cell cycle	MB	
**miR-199b-5p**	↓ in HES1 overexpressing MB	HES1	↑ compared to normal brain tissue	Suppression	Suppresses proliferation/cell cycle	MB	Garzia L, Andolfo I et al. [69]
**miR-17 ~ 92**	↑ in MB and Embryonal Neural Tissue	Sonic Hedgehog signaling (Ptch1, p53, Ink4c, Nmyc)	↑ compared to normal brain tissue	Promotion	Promotion	MB	Uziel T, Karginov FV et al. [63] / Northcott PA, Korshunov A et al. [52]
**miR-2**							
**miR-19a**							
**miR-20**							
**miR-106a–92**							
**let-7 g**	↓ in Classic, Anaplastic, Desmoplastic MB				Distinguishes Types	MB	Ferretti E, De Smaele E et al. [76]
**miR-19a**							
**miR-191**	↑ in Classic, Anaplastic, Desmoplastic MB				Distinguishes Types	MB	
**miR-106b**							
**miR-10b**	Mixed pattern of expression	ERBB2 (ErbB)	Both ↓ ↑		Distinguishes between Over- and Underexpressing MBs	MB	
**miR-125b**							
**miR-135a**							
**miR-135b**							
**miR-153**							
**miR-199b**							
**miR-128-1**	Mixed pattern of expression	CMYC	Both ↓ ↑		Distinguishes between Over- and Underexpressing MBs	MB	
**miR-128-2**							
**miR-181b**							
**miR-30b**	↑ in MB at 8q24.22-q24.23	KHDRBS3	↑ compared to normal brain tissue		Correlates	MB	Lu Y, Ryan SL et al. [80]
**miR-30d**							
**miR-17**	Mixed pattern of expression				Distinguishes between MB tumor tissue and adjacent Normal tissue	MB	Liu W, Gong YH et al. [81]
**miR-99a**							
**miR-100**							
**miR-106b**							
**miR-204**							
**miR-218**							
**miR-29a**							
**miR-29c**							
**miR-128a**							
**miR-379**							
**miR-127-3p**							
**miR-9**							
**miR-9**							
**miR-214**							
**miR-34a**	↓ compared to normal brain tissue	CMET	↑ compared to normal brain tissue	Suppression	Suppresses proliferation/cell cycle	MB	Guessous F, Zhang Y et al. [82]
		NOTCH1	↑ compared to normal brain tissue	Suppression	Suppresses proliferation/cell cycle	MB	
		MACE-A	↑ compared to normal brain tissue	Suppression	Enhances Chemosensitivity	MB	Weeraratne SD, Amani V et al. [84]
		TP53	↓ in MB	Indirect promotion		MB	
**miR-128a**	↓ compared to normal brain tissue	BMI1	↑ compared to normal brain tissue	Suppression	Suppresses tumor growth through senescence	MB	Venkataraman S, Birks D et al. [77]/Venkataraman S, Alimova I et al. [78]
**miR-142-5p**	↓ compared to normal brain tissue			Promotion	Probable tumor enhancement	MB	Birks D, Barton VN et al. [85]
**miR-25**							
**miR-193a**	↑ compared to normal tissue	ATOH1, MYCN, TRPM3			Differentiation/Proliferation	MB	Gokhale A, Kunder R et al. [68]
**miR-183**							
**miR-224**							
**miR-182**							
**miR-452**							
**miR-204**							
**miR-365**							
**miR-135a**							
**miR-23b**							
**miR-148a**							
**miR-27b**							
**miR-24**							
**miR-146b**							
**miR-335**							
**miR-98**							
**mIR-199a**							
**miR-92**							
**miR-565**							
**miR-135b**							
**miR-193b**							
**let-7c**		LEMDE1, KHDRBS2, TRPM3,GRM8					
**miR-135b**							
**miR-193b**							
**miR-7**							
**miR27b**							
**miR-204**	↓ compared to normal brain tissue						
**miR-153**							
**miR-410**							
**mIR-487b**							
**miR-433**							
**miR-127**							
**miR-21**	↑ compared to normal tissue				Suppression impedes migration	MB	Grunder E, D’Ambrosio R et al. [73]
**miR-128**	↑ compared to normal tissue	Musashi1	↑ compared to normal brain tissue			MB	Vo, Qiao et al. [74]
**miR-34a**							
**miR-101**							
**miR-138**							
**miR-128**							
**miR-137**							
**miR-183 ~ 96 ~ 182**	↑ compared to normal tissue	PI3K/AKT/mTOR	↑ compared to normal brain tissue		Cell survival, proliferation, migration	MB	Weeraratne SD, Amani V et al. [84]
**miR-33b**	↓ compared to normal brain tissue	CMYC			Its absence promotes MB, when expressed represses MB	MB	Takwi AA, Li Yet al. [79]
**miR-383**	↓ compared to normal brain tissue	PRDX3			Its absence promotes MB, when expressed represses MB	MB	Li KK, Pang JC et al. [88]
**miR-494**	↑ compared to normal tissue	SDC1			promotes angiogenesis	MB	Asuthkar S, Velpula KK et al. [89]
**miR-218**	↓ compared to normal brain tissue	SH3GL1		Suppression	Cell survival, proliferation, migration	MB	Shi J, Yang L et al. [90]
**miR-193a-3p**	↑ compared to normal tissue	WNT signaling pathway (CTNNB1, WIF1, DKK2, MYC)		Promotion	Cell survival, proliferation, migration	MB	Kunder R, Jalali R et al. [54]
**miR224**							
**miR-148a**							
**miR-23b**							
**miR-365**							
**miR-let-7f-1**	↓ compared to normal brain tissue				regulator of cis-platin resisance	MB	Pannuru P, Dontula R et al. [92]
**miR-31**	↑ compared to normal tissue	MCM2	↑ compared to normal brain tissue	Suppression	Inhibition of Chromatin remodelling	MB	Jin Y, Xiong A et al. [91]
**miR-517c**	↑ compared to normal tissue	WNT signaling pathway (API5, BAD, TRADD, SOX11, NR2F1 NKX2-2, FGF13, FGFr3, SALL4)		Promotion	Cell survival, proliferation, migration	PNET	Li M, Lee KF et al. [102]
**miR-520 g**	↑ compared to normal tissue	NKX2-2,OLIG1		Promotion			
**miR-let-7f-1**	↓ compared to normal brain tissue	LIN28, IGF/PI3K/mTOR		Promotion	Cell survival, proliferation, migration	PNET	Spence T, Perotti C et al. [105]
**miR-221**	↑ compared to normal tissue	p27^Kip1^	↓ in ATRT	Suppression	Tumor progression	ATRT	Sredni ST, de Fátima Bonaldo M et al. [111]
**miR-222**	↑ compared to normal tissue	p27^Kip1^	↓ in ATRT				Sredni ST, de Fátima Bonaldo M et al. [111]
**miR-517c**	↑ compared to normal tissue					ME/ EB	Nobusawa S, Yokoo H et al. [109]
**miR-520 g**	↑ compared to normal tissue						
**miR-371**	↑ compared to normal tissue						
**miR-372**	↑ compared to normal tissue						
**miR-373**	↑ compared to normal tissue						
**miR-142-5p**	↑ compared to normal tissue					ATRT	Birks DK, Barton VN et al. 2010
**miR-25**							
**miR-520b**							
**miR-629**							
**mIR-498**							
**miR-373**							
**miR-140**	↓ compared to normal brain tissue					ATRT	Birks DK, Barton VN et al. 2010
**miR-let-7b**							
**mIR-139**							
**mIR-153**							
**miR-376b**							
**miR-let-7a3**	↓ compared to normal brain tissue	HMGA2	↑ compared to normal brain tissue			ATRT	Zhang K1, Gao H et al. [10]
**miR-let-7b**							

## Medulloblastomas

Medulloblastomas (MB) are the most common malignant pediatric neoplasms of the CNS and represent >20% of all pediatric brain tumors. According to WHO classification, MB grade IV is a malignant embryonal tumor of the cerebellum with a preferential manifestation in children, predominantly neuronal differentiation and an inherent trend to metastasize via cerebrospinal fluid (CSF) pathways [46]. Staging systems for MBs based on clinical parameters including patient age, metastatic stage and pathological variants are still widely employed in clinical practice. However, they do not sufficiently reflect the true clinically and biologically heterogeneous nature of these neoplasms [47-51]. The group of MB consists of: a) classical b) desmoplastic/nodular c) MB with extensive nodularity d) anaplastic large cell MB. Following the WHO Classification, two new variants are recognized; medulloblastoma with extensive nodularity and anaplastic MB. Therefore, the descriptive terms MB with rhabdomyoblastic differentiation and MB with melanotic differentiation have been proposed.

Although aggressive multimodal therapy has improved the prognosis for children with MB, nearly half of all patients will eventually die from progressive tumors. Moreover, survivors often suffer significant treatment-related morbidities, including neurocognitive deficits related to radiation therapy. New insights into the pathogenesis of these tumors are therefore sorely needed. Gene-based research has identified two subgroups of MBs, one associated with mutated genes within the Hedgehog pathway and the other associated with altered Wingless-type MMTV integration site family (WNT) pathway genes [52]. It is noteworthy that children with WNT MBs have improved survival [53]. Also, WNT MBs have manifested significant over-expression of miR-193a-3p, miR224, miR-148a, miR-23b and miR-365 [54]. Amplifications of *MYC* and the transcription factor *OTX2* [55], mutations in *TP53* [56], and a number of chromosomal alterations have also been identified in MBs. These discoveries have assisted to define the pathogenesis of MBs and have improved the ability to identify patients who might benefit from therapies targeting these pathways. However, most MB patients do not have alterations in these genes and the compendium of genetic alterations causing MB remains to be determined.

### MicroRNAs and Medulloblastomas

The first report on miRNA role in medulloblastoma was published by *Pierson et al.* [58], in which it was found that CDK6 was regulated by miR-124, a tumor suppressor [57], and miR-24a was found to be a negative regulator of SLC16A1 [58,59]. Later on, a similar report confirmed the interaction of another miRNA; miR-22, with another member of this family of proteins, the PAPST1 (SLC35B2), indicating a constant down-regulation in MBs and a potential inhibitory role in disease progression [60]. In addition, CDK6 was shown to be regulated by miR-129, which functioned as a potential tumor suppressor [61]. Further on, it has been reported that miRNAs interact with the Hedgehog pathway. In particular, in a previous study it was found that MB tumors manifest two categories of neoplasms based on the levels of the GLI1 gene and respective protein that is those with high levels and those with low levels. *Smoothened* (Smo) is an effector of Gli1, which is activated and starts its transcriptional activity. Both proteins are mediators of the *Hedgehog* pathway, whose hyperactivity has been reported in MBs, and thus its regulation could be of significance. In that sense, it was found that miR-326, miR-135a, miR-135b, miR-125b, miR-103, miR-203, miR-338, miR-324-5p, miR-100, miR-153, miR-324-5p and miR-331 were regulating Smo and Gli1, respectively [62]. The interesting finding was that those miRNAs were downregulated in almost all MBs and their activation led to MB cell proliferation inhibition and growth [62]. Similarly, a second report showed the significance of another miRNA in MB; miR-17 ~ 92 cluster. It appeared that this miRNA was overexpressed in MBs and interfered with the *Hedgehog* pathway [52,63,64], while its silencing inhibited MB progression [65,66]. At the same time, it was found that miR-182 participated in non-*Sonic Hedgehog* MBs, promoting migration [67]. Several miRNAs have been reported to be associated with the Sonic Hedgehog and WNT pathways. Those are summarized in Table 2, where MB tumors have been separated into four groups, according to the report, with the respective up- or down-regulated miRNAs and some of the respective genes (second row) [68].

**Table 2:** **miRNAs implicated in MB and the WNT, SHH signaling pathways** Gokhale et al. [68]

	**A**	**B**	**C**	**D**
	**WNT signaling**	**SHH signaling**	**Proliferation differentiation**	**Differentiation/Proliferation**
	**WIF1, GABRE, CTNNB1**	**ATOH1, MYCN, HHIP, PTCH1, GLI2, TRPM3, miR-135b, miR-204**	**LEMDE1, KHDRBS2, miR-135b**	
			**TRPM3, miR-204**	**GRM8**
**Up-regulated**	miR-193a	miR-199a	miR-135b	let-7c
	miR-183	miR-92	miR-193b	miR-7
	miR-224	miR-565		miR27b
	miR-182			
	miR-452			
	miR-204			
	miR-365			
	miR-135a			
	miR-23b			
	miR-148a			
	miR-27b			
	miR-24			
	miR-146b			
	miR-335			
	miR-98			
**Down-regulated**	miR-376a	miR-135b	miR-204	
	miR-127	miR-204	miR-153	
	miR-134	miR-153	miR-410	
	miR-181d		mIR-487b	
	miR-9		miR-433	
	miR-181c		miR-127	

Another molecule of interest in MBs appeared to be HES1. Persistent expression of bHelix-Loop-Helix (bHLH) HES1, the principal *Notch*-responsive gene, prevented both migration of neural progenitor cells out of the ventricular zone and expression of neuronal markers [69] as well as epigenetic silencing of miR-9 was also associated with HES1 oncogenic activity [70]. In the same report, it was shown that miR-199b-5p impairs MB progression, when overexpressed, and reduced proliferation [69]. HES1 has been found to be also regulated by miR-199b-5p along with CD15 [71]. In a recent review, it has been reported that miRNAs have been found to participate in a variety of brain tumors. In particular, in Glioblastoma Multiforme (GBM), several miRNAs such as miR-10b, miR-21, miR-124 and miR-128-1 were reported to be deregulated, as well as glioma stem cells were found to upregulated miR-16, miR-107, miR-185, miR-425 and miR-486 [59,72]. Further on, miR-21 when suppressed inhibited the potential MB migration [73] and miR-128 regulated the RNA-binding protein *Musashi1* (expression of this gene has been correlated with the grade of the malignancy and proliferative activity in gliomas and melanomas) along with miR-34a, miR-101, miR-128, miR-137 and miR-138 [74]. It has also been shown that miR-191, mIR-106b and miR19a could distinguish between classic, anaplastic and desmoplastic MBs. For miR-106b, it has also been reported in a subsequent study that it was up-regulated in MBs and interacted directly with PTEN, a critical signaling molecule in disease progression [75]. Also, miR-10b, miR-125b, miR-135a, miR-135b, miR-153 and miR-199b were clustered and thus able to differentiate between ERBB2 high and low expression in MBs, while miR-128-1, miR-128-2, and miR-181b were able to distinguish between CMYC high and low expression MBs [76]. MiR-128a was also reported to inhibit MB growth, yet through senescence, by targeting BMI1, a gene which is a component of a Polycomb group (PcG) multiprotein PRC1-like complex, which is required to maintain the transcriptionally repressive state of many genes, including Hox genes, throughout development. It is directly involved in embryonal tumors and erythroplakia [77,78]. The cMYC was also found to correlate with miR-33b, which when expressed represses MB promotion. The 17p11.2 locus is usually missed in MBs and it is the location of miR-33b [79].

Another miRNA studied was miR-30b and miR-30d, which appeared to be upregulated in MBs with the amplification 8q24.22-q24.23, along with KHDRBS3 gene; an RNA-binding protein that plays a role in the regulation of alternative splicing and influences mRNA splice site selection and exon inclusion [80]. One of the few microarrays miRNA analyses performed in 2009 by Liu et al. showed the potential differences between MB tissue and adjacent normal tissue. The miRNAs reported are of interest since it is known that tumors interact with their microenvironment, thus those miRNA could be potential targets of MB functionality and metastasis [81]. Returning to the individual miRNA roles, an additional report showed that miR-34a was a potential tumor suppressive gene and interacted with CMET and NOTCH1. Both, are considered known genes for their role in tumorigenesis and tumor ontogeny [82,83]. Additionally, the same miRNA has been found to be involved in chemosensitivity through regulation of MAGE-A and p53 in MBs [84], as well as regulating the RNA-binding protein *Musashi1* [74]. When comparing several CNS tumor types, a report found two miRNAs that were upregulated in all types. Those included miR-142-5p and miR-25, which appeared to be upregulated in GBM, MB AT/RT, EPN and PA. Probably, those miRNAs could be regarded as common regulators for CNS tumors [85]. In some most aggressive types of MD, associated with MYC amplification, the group of miR-183 ~ 96 ~ 182 cluster has been found to be implicated in MB survival, proliferation and migration through the PI3K/AKT/mTOR signaling axis [86,87]. Another recently reported miRNA, miR-383 was down-regulated in MBs, while when ectopically expressed inhibited growth and MB survival through PRDX3 regulation [88].

The role of miRNAs is being more and more elucidated as novel mechanisms are being revealed. In a very recent report, it was proposed that ionizing radiation (IR)-induced MMP-9 enhances SDC1 shedding, corroborating to tube-inducing ability of MB cells. Furthermore, it has been reported that tumor angiogenesis is associated with higher MMP-9-SDC1 interactions on both the cell surface and extracellular medium, revealing that the existence of a novel regulatory mechanism where MMP-9 drives the suppression of miR-494, resulting in enhanced SDC1 shedding and angiogenesis. Hence, it has been found that MMP-9-specific shRNA treatment of mouse intracranial tumors resulted in elevated expression of miR-494. Further analysis showed that SDC1 mRNA is a direct target of miR-494 [89]. Another miRNA identified was miR-218, a tumor suppressor, which is down-regulated in MB directly targeting SH3GL1, a gene whose overexpression may play a role in leukemogenesis, and the encoded protein has been implicated in acute myeloid leukemia as a fusion partner of the myeloid-lymphoid leukemia protein [90]. Two novel reports have shown that miR-31 suppresses MB by inhibiting MCM2 [91] and lastly, that miR-let-7f-1 is a regulator of cis-platin resistance in MB cells [92].

Up to this point, it is apparent that the knowledge gathered has been essential for understanding the role of miRNAs in MB. Yet, it is also quite obvious that further research is necessitated in order to comprehend the complicated mechanisms of MB progression and oncogenesis.

## Primitive Neuroectodermal Tumors (PNETs)

Central nervous system primitive neuroectodermal tumors (PNETs) are a heterogeneous group of tumors occurring predominantly in children and adolescents, arising in the cerebral hemispheres, the brain stem and spinal cord. CNS PNETs are composed of undifferentiated or poorly differentiated cells, which may display divergent differentiation along neuronal, glial or ependymal cell lines. Features common to all CNS PNETs include early onset and aggressive biological behavior. The term CNS PNET encompasses: a) CNS supratentorial PNET, (CNS PNET) embryonal tumors composed of undifferentiated or poorly differentiated neuroepithelial cells that occur at any extracerebellar site b) CNS neuroblastomas, tumors with only neuronal differentiation c) CNS ganglioneuroblastomas when ganglion cells are also present d) medulloepitheliomas, tumors with features of embryonal neural tube and e) ependymoblastomas. Moreover, an unusual PNET, called embryonal tumor with abundant neutrophil and true rosettes occurring in the cerebrum of young children, is mentioned as a provisional entity.

The cytogenetics of PNETs are considered better understood than the rest pediatric CNS tumors, with several recognized, nonrandom chromosome abnormalities. The most frequent observed in 30–40% of cases, is loss of 17p, usually through the formation of an isochromosome of 17q [93,94]. Additional regions of genetic imbalance also have been demonstrated using microsatellite analysis [95] and CGH [96,97] with some studies suggesting genetic differences between intratentorial and supratentorial PNET [98,99]. In general, investigations on supratentorial PNETs are infrequent, however despite the small number of genetic studies, CNS/suprantetorial PNET show different genetic alterations than medulloblastoma, which argues against the previously proposed theory; PNET concept, for common histogenesis. CNS supratentorial PNETs usually show no loss of 17p, i17q and patched (*PTCH*) gene mutations, which characterize medulloblastomas. In contrast, supratentorial PNETs express neurogenic transcription factors of the NeuroD family and HASHI, a neurogenic transcription factor not detected in MB. It is notable that children with supratentorial PNETs, especially those less than 2 years old, have a dismal overall 5 year survival than children with medulloblastoma, suggesting that there might be additional, yet unknown constitutional genetic aberrations underlying the pathogenesis of PNETs.

### MicroRNAs and PNETs

There are not many reports concerning this type of embryonal tumor with miRNAs. One report has found that in an embryonal tumor cell line miR-let-7 is linked to mTOR signaling and the miRNA remains underexpressed and it is upregulated in LIN28 knockdown cells along with IGF/PI3K/mTOR pathway signaling silencing [100]. This finding was important since LIN28A is considered to be a diagnostic marker for embryonal tumors and thus its expression could be of critical significance for disease progression [101]. One report that dealt with PNETs has mentioned that miR-517c and miR-520 g promoted oncegenicity interacting with WNT signaling [102]. As in the case of MBs also in PNETs similar signaling pathways are implicated hinting towards a common machinery in CNS Embryonal tumors, which remains to be elucidated. The functions of miRNA in neuroectodermal tumors are not limited to the CNS but numerous reports have reported a role in neuroblastoma or even Ewing Sarcoma /PNET [103,104].

## Other embryonal tumors

### Medulloepitheliomas (ME)

According to WHO 2007, medulloepithelioma (ME) grade IV is a rare malignant embryonal neoplasm affecting young children characterized by tubular, papillary and trabecular arrangement of neoplastic cells recapitulating the features of embryonal neural tube. MEs are affecting children between 6 months and 5 years with 50% occurring during the first 2 years of life. Congenital cases and cases occurring beyond the first decade have been reported. The periventricular area is the most common site in the cerebral hemispheres involving mainly the temporal and parietal lobes. ME may arise intraventricularly, in the sellar region, *cauda equine* and presacral area. Intraorbital ME rarely metastasize and have a favorable prognosis, while optic nerve tumors carry an intermediate prognosis between intraorbital and cerebral tumors. The differential diagnosis of ME includes: a) ependymoblastoma, characterized by predominant ependymoblastic rosettes, b) choroid plexus carcinomas, which are strongly Cytokeratin + without the characteristic neuroepithelium, c) immature teratoma that contains tissues from the three germ layers and d) AT/RT, which shows loss of nuclear INI1 protein. ME is considered a very aggressive neoplasm, since most children are dying within a year of diagnosis. Histologically MEs recreate the features of embryonal neural tube. They are composed of a) tubular, papillary and trabecular arrangement of neuroepithelium with a limiting external membrane and b) sheets of undifferentiated cells and areas with divergent differentiation. The diagnostic feature of ME is the pseudostratified neuroepithelium arranged in papillary and tubular structures composed of cuboidal and columnar cells with the nuclei perpendicular to the inner/outer surface showing nucleoli and luminal mitoses. Immunohistochemistry reveals in the neuroepithelial component expression of nestin and vimentin mainly confined to the basal area of the stratified epithelium. In neuronal areas, variable expression of Synaptophysin, Neurofilaments, EMA and cytokeratins has been observed, while there is no detection of glial fibrillary acid protein (GFAP), S-100 and neuron specific enolase (NSE). In the areas distinct from the neuroepithelium, variable expression of synaptophysin, neurofilaments and *MAP2* is seen in neuronal areas, while *GFAP* expression is variable/limited in undifferentiated areas, while increasing in areas of astrocytic differentiation. The histological/immunohistochemical resemblance of ME with embryonal neural tube favors its derivation from a progenitor in the subependymal area. The overall picture regarding the genetic profile is somewhat vague and therefore needs to be verified.

#### MicroRNAs and MEs

As in the case of PNET, a basic player in ME is LIN28, which co-existing with a C19MC amplification consists of a distinct histogenetic, diagnostic and therapeutic entity as recently reported [105]. The presence of C19MC amplification has been also highlighted for embryonal tumors as it has been shown that embryonal tumor oncogenesis is driven genetically by the fusion of TTYH1 to C19MC involved in fetal neural development [106].

### Ependymoblastomas (EB)

According to WHO 2007, ependymoblastoma Grade IV is a rare embryonal neoplasm affecting mainly infants and young children. Ependymoblastomas are aggressive tumors with craniospinal dissemination and fatal outcome in 6 months to 1 year. Histologically, they are primitive neuroectodermal tumors with increased cellularity and characteristic ependymoblastic rosettes. The rosettes are multilayered and form concentric rings around a well-defined central lumen. Their nuclei are pushed away from the lumen and the apical surface of the cells show blepharoplasts and forms a distinct internal membrane. The outer layer of the cells merges with the adjacent undifferentiated neuroepithelial cells. Immunohistochemistry in a limited number of cases reveals expression of S-100, vimentin, cytokeratin, GFAP, carbonic anhydrase isoenzyme II, rarely neurofilaments 68.160.200 Kda. The tumors are presumed to derive from periventricular primitive cells. The term ependymoblast implies an incompletely differentiated cell with glial-ependymal features and immature characteristics. They are associated with inferior prognosis but sustained remissions have been achieved after multimodal treatment [107]. Moreover, they are considered diagnostically challenging subtypes of embryonal tumors, whose genetic features remain unknown. Previous work suggested that ependymoblastomas show distinct and fairly consistent chromosomal aberrations [108]. However, the relative contributions of the different genetic changes to disease pathogenesis need to be verified in larger studies.

#### MicroRNAs and EBs

This is also a tumor type that manifests the 19q13.42 amplification and is linked to the previously mentioned miRNA cluster; C19MC, with specifically the miR-371-373 being up-regulated [109]. In addition, the same study manifested the up-regulation of miR-517c, miR-520 g, miR-371, miR-372 and miR-373 in ependymoblastoma and medulloepithillioma tumors.

### Atypical teratoid rhabdoid tumor /rhabdoid tumor predisposition syndrome (AT/RTs)

Atypical Teratoid Rhabdoid Tumor /Rhabdoid Tumor predisposition syndrome (RTPS) is a disorder characterized by an increased risk to develop malignant rhabdoid tumours (MRT) generally due to constitutional loss or inactivation of one allele of deletion *INI1/hSNF5/SMARCB1* gene located on chromosome 22q11.2. Children with multiple MRT or with affected siblings or with other relatives are almost certainly afflicted by the syndrome, while familial cases are rare. Atypical Teratoid Rhabdoid tumour (AT/RT) is a highly malignant CNS tumour predominantly in young children, with a male preponderance, typically containing rhabdoid cells often with primitive neuroectodermal cells and divergent differentiation along epithelial, glial, neuronal, mesenchymal-like lines. AT/RT represents 1-2% of paediatric brain tumours and accounts for at least 10% of CNS tumours in infants, due to the predominance in children younger of 3 years old. It can be supratentorial, especially in cerebral hemispheres less frequently in the ventricular system, supraselar region and pineal gland, or intratentorial, especially in the cerebellar hemispheres, cerellopontine angle, brain stem mainly in children younger than 2 years of age.

Histologically, the hallmark of AT/RT is heterogeneity. Rhabdoid cells are the characteristic features in many cases, corresponding to cells with eccentric nuclei containing fine chromatin and a prominent eosinophilic nucleolus, well-defined abundant eosinophilic cytoplasm with a globular inclusion. Rhabdoid cells are characterized by morphological variation, being large with less atypic, abundant finely granular cytoplasm or cytoplasmic vacuolization. Nests, sheets or a jumbled appearance of rhabdoid cells is observed, while they represent the predominant cells only in the minority of AT/RT. Most neoplasms have variable components with PNET-like, mesenchymal and epithelial features.

Immunohistochemistry reveals expression of various markers reflecting the polyphenotypic differentiation of AT/RT. More specifically, it shows strong vimentin reactivity as well as the epithelial membrane antigen, smooth muscle actin, GFAP, cytokeratin, desmin, S-100 protein and neuron specific enolase. The above markers are variably expressed in areas with different histological patterns. Pathogenesis of AT/RT remains unknown. Based on commonly found *INI1* gene inactivation, it is considered that lack of INI1 protein might play a significant role in the process. In general, prognosis of AT/RT is dismal while there are no protocols aimed specifically for AT/RT. The vast majority of children receive platinum-based and alkylator-based regimens which are commonly recommended for brain tumours in infants. Therefore, it is essential to identify novel therapeutic targets.

#### MicroRNAs and AT/RTs

In a recent report, it was found that two miRNAs; miR-221 and miR-222 are participating in AT/RT ontogenesis. In particular, it was found that those miRNAs are potent regulators of p27^Kip1^, a tumor suppressor molecule, member of the CDK family of inhibitors [110]. Those miRNAs have been found to be up-regulated in AT/RTs thus inhibiting the p27^Kip1^ function and allowing the tumor to progress [111]. In another report, it was reported that miR-142-5p and miR-25 were up-regulated in AT/RTs as compared to normal tissue, while miR-129 was down-regulated in AT/RTs as compared to normal tissue [85]. Collectively, in the same report the up-regulated miRNAs in AT/RTs included, miR-520b, miR-629, miR-221, miR-448 and miR-373, while the down-regulated miRNAs were miR-140, miR-let-7b, miR-139, miR-153 and miR-376b [85].

A different study that compared the expression levels of miR-517c, miR-520 g, miR-371, miR-372 and miR-373 showed that there was no amplification of the genes as compared to ependymoblastoma and medulloepithilioma [109]. Another recent report studied the differences between AT/RT and Rhabdoid Tumors of the Kidney (RTK) trying to identify miRNAs that would separate the two tumor types. It was found that there were no miRNAs that could distinguish between the two tumor types, probably due to the fact that both are of the same origin, irrespectively of the focal point [112]. MiR-let-7a3 and miR-let-7b were found to be down-regulated in AT/RTs and at the same time significantly reversibly correlated to HMGA2, a gene that encodes a protein that belongs to the non-histone chromosomal high mobility group (HMG) protein family. HMG proteins function as architectural factors and are essential components of the enhancesome. This protein contains structural DNA-binding domains and may act as a transcriptional regulating factor. Identification of the deletion, amplification, and rearrangement of this gene that are associated with myxoid liposarcoma suggests a role in adipogenesis and mesenchymal differentiation [25]. In a most recent report on AT/RTs, it was mentioned that a subpopulation of CD133(+) cells isolated from AT/RT tumors is present, having cancer stem-like and radio-resistant properties. It was shown that expression of miR142-3p was lower in AT/RT-CD133(+) cells than in AT/RT-CD133(−) cells. miR142-3p overexpression significantly inhibited the self-renewal and tumorigenicity of AT/RT-CD133(+) cells. On the contrary, it was found that silencing of endogenous miR142-3p dramatically increased the tumor-initiating and stem-like cell capacities in AT/RT cells or AT/RT-CD133(−) cells and further promoted the mesenchymal transitional and radio-resistant properties of AT/RT cells [113]. As opposed to MB tumors, AT/RTs are less studied and there is still a lot to be learned about their underlying biology and pathogenesis mechanisms.

### Microrna detection-current methodologies

Discussing miRNAs is one thing, but detecting them is another. Considering the fact that miRNA abundance is at low percentages within the total RNA quantity, it is necessary that detection methods should be accurate and precise [114]. The good news is that such methods exist and are extremely helpful in detecting miRNA expression variations. In brief, methodological approaches could be separated into the following categories: Polymerase Chain Reaction (PCR)-based methods, which include both simple PCR as well as quantitative Real-Time PCR (RT-qPCR), microarray based methodologies, Northern blot analyses, *in situ* hybridization and last but not least Next Generation Sequencing (NGS) methodologies [115]. Each of the aforementioned methods has its own benefits and drawbacks. We could make a brief discrimination if we would say that they can be divided into two major categories: those that can detect a small number of miRNAs and those that can detect large numbers of miRNAs simultaneously. To the first category belong the PCR methodologies (even though the use of plates in the case of RT-qPCR could classify them to the high-throughput methods, it is still low in number as compared to the other methods), blotting and *in situ* hybridization. To the other end microarray-based platforms and NGS are capable of detecting and examining the complete “*miRNome*” in an experiment, a fact that makes them very attractive for discovery-based investigations. On the other hand, RT-qPCR affords the gold standard in miRNA detection and quantification. This method is frequently required following a high-throughput experiment in order to verify the obtained results. qRT-PCR is difficult to be used in high-throughput analysis [116] and it is also considered an expensive technique, however it is less time consuming than microarrays and NGS and does not require complex data processing by biostatisticians [117]. At the same time, Northern blot analysis remains an undisputable tool in gene expression validation, yet it has a drawback involving the time consumed, the poor sensitivity [118] and the amount of available miRNA in a given sample [115]. The latter detail leads to poor sensitivity in miRNA detection and can be surpassed only by the excessive use of sample, which is not always possible. *In situ* hybridization methods although provide sensitive miRNA detection, are considered semi-quantitative in contrast to capillary electrophoresis techniques which allow simple quantitative analysis with high resolving power [118]. Last but not least, the NGS methods are becoming a powerful tool in the detection of miRNA expression since they cannot only detect the abundance of miRNA in a sample but also the presence of novel, unknown miRNAs as well as miRNA mutations. It is very probable that this methodology will replace all previous, with time, both in research as well as in the clinical praxis. Its cost remains high but it is decreasing as novel plarforms are developing [117].

### Therapeutic applications

MicroRNAs are considered attractive therapeutic tools for at least two reasons; they are low toxicity for their endogenous expression and due to their multi-targeting properties. Regulation of miRNA expression can be achieved in several ways. One approach to design synthetic miR-like small RNA molecules, which would suppress the production of proteins known to be involved in certain disease conditions. An additional strategy would be to target endogenous miRs either by mimicking the effects of miRs that are pathologically underexpressed in disease or by antagonizing the effects of miRs that are abnormally overexpressed in disease.

Antagomirs are of major interest since they are delivered to almost all tissues, with the exception of the brain, following injections systemically or locally. Of note, according to *Wang* et al. [119], this miRNA feature might bring about ‘off-target’ side-effects [119]. Nevertheless, antagomirs against miR-21 have been successfully used to inhibit fibrosis of heart and attenuate cardiac dysfunction [120]. Furthermore, a miR-133a directed antagomir has previously been employed to prevent hypertrophy [121]. Even though, antagomirs have proved extremely valuable in the laboratory, yet there is a long way until miR-based therapeutic strategies are implemented in human disease. Most importantly, therapeutic delivery of miR142-3p in AT/RT cells effectively reduced its lethality by blocking tumor growth, repressing invasiveness, increasing radiosensitivity, and prolonging survival time in orthotropic-transplanted immunocompromised mice.

Blocking oncogenic miRNAs can be achieved by the use of antisense oligonucleotides, miRNA sponges, miR-masks and small RNA inhibitors [122]. The most straightforward method to inhibit miRNA repression of protein expression uses anti-miRNA oligonucleotides (AMOs) to specifically restrain interactions between the miRNA induced silencing complex (RISC) proteins and the miRNA or miRISC and its target mRNAs [87,123]. An additional approach is to block the activity of a specific miRNA using a competitive inhibitor known as a miRNA sponge or target mimic, which contains binding sites for the miRNA either in a non-coding transcript or in the 3’ untranslated region (UTR) of a reporter gene [124]. Small-molecule miRNA inhibitors can regulate miRNA expression at the transcriptional level [122].

Enhancing of miRNA expression requires more factors to be taken into account than blocking a specific miRNA. Normally, increasing the expression of a molecule requires the repression of a blocking precursor or the direct addition of the desired molecule. Both of these techniques are difficult to achieve at least *in vivo*. This type of application is called miRNA replacement therapy through the use of miRNA *mimics*. In order to achieve similar biological function as the naturally produced miRNAs, mimics should enter the RISC complex and affect miRNA target mRNAs [87,123].

Several reports have shown that it is possible to introduce/deliver therapeutic miRNAs through viral vector-based systems in tumor tissues *in vivo* [115,125-128]. Additionally, several studies have reported the insertion of miRNAs or the enhancement of their expression *in vitro* [129,130]. In general, materials for miRNA and anti-miRNA delivery include lipid-based systems, polyethylenimine-based systems, dendrimers, poly(lactide-co-glycolide) particles and other non viral delivery systems including naturally occurring polymers such as chitosan or atelocollagen, among others [87,123]. Additionally, hemispheres and hydrogels have also been developed as gene delivery vehicles [122].

So far, significant advances have been made in the development of miRNA-based therapies for cancer and other human diseases. Mirna Therapeutics™ has initiated a Phase 1 clinical study with. MiRX34, which is a liposome-formulated miRNA mimic of the tumor suppressor miR-34, for use in patients with liver cancer or metastatic cancer with liver involvement. This study is currently recruiting participants to evaluate MRX34 safety [131].

Certainly, there are several limitations in the use of miRNAs as therapeutic targets. Some of these limitations apply not only to miRNAs but to other therapeutic targets including proteins or genes and some others apply miRNAs alone. A general rule of target investigation and usage is the lack of knowledge on basic mechanisms. Since, biological systems tend to be extremely complicated the time of unraveling their unknown role is disproportionate to the need for therapies. Especially for miRNAs, their biology is still largely unknown and the question whether the aberrant expression of such a molecule is due to the disease or it takes place during a physiological process still remains unanswered. Yet, up to date, this is a global issue in target discovery that has not been dealt with. Of note though, miRNAs possess several advantages as compared to other molecules. They are small and their targets (as far as we know) are genes [122]. Blocking or enhancing a miRNA could probably produce avalanche effects and has an optimum result with respect to therapy. However, effective delivery and selective target delivery still remain an issue, since their side- effects could be disproportionate to their size.

Especially, in the case of CNS tumors there is another obstacle that should be overcome, that is the Blood–brain-Barrier. This particularity of the brain requires the use of sophisticated techniques in drug delivery but also calls an extreme caution towards the adverse effects that miRNAs could have in the brain [132]. In the discussion about therapy, a very important factor should not be omitted. This includes the use of miRNAs as prognostic markers for chemotherapy and radiotherapy. Several reports have highlighted this aspect as it has been shown that the over- or down-regulation of specific miRNAs is linked to sensitivity to classical anti-cancer therapies [115,133-136]. This fact makes miRNAs even more promising markers for cure since their dual role of either being the medication *per se* or the prognostic factor gives them great value for further investigations.

### Challenges in microrna biology and use

It is a general belief that the ultimate end-point in miRNA discovery is their use for clinical purposes, either prognostic or therapeutic. Yet, besides those noble tasks, miRNAs could serve as amplifiers of knowledge in more basic scientific questions such as evolution and biological mechanics *per se,* that understand their role in the preservation of life. It is not overstated if we say that the discovery of miRNAs has added another unknown variable in the equation of biological systems. From that point of view, efforts should continue towards the discovery of basic mechanisms underlying miRNA functions and role.

On the other hand, clinical applications still have some obstacles to overcome. The first comes from our previous point that referred to the lack of basic knowledge on miRNA biology. The second comes from application issues such as effectiveness of delivery, off-target effects, side-effects and final accuracy of treatment. To our view, an effective way of clinical use, towards therapy, would the discovery of up-regulated miRNAs in tumors and its subsequent blocking rather than the mimicking of a miRNA. On the other hand, recent development in miRNA discovery has given very promising results towards their use as prognostic factors and the subsequent use in improving classical therapies.

## Concluding remarks

In summary, even if miRNAs appear as promising therapeutic targets, there is still a huge gap between mi-RNA basic research and application to clinical settings. Drug efficacy and toxicity studies are warranted to enhance our knowledge about the impact of miRNA-directed therapeutics before proceeding to clinical treatments. Therefore, advancing our understanding regarding the role of miRNAs expression profiles and their involvement in translational regulation holds a promising future for pharmacogenomics. Another comment that we could make on the mechanistic nature of miRNAs is that a basic observation we have done was that similar miRNAs participate in similar signaling pathways and in different neoplasmatic types. This is a hint towards a probable common mechanistic regulatory mechanism in CNS tumors, with great implications for disease prognosis and therapy. Therefore, it is justified to say that miRNAs are currently the *grand known unknown*.
